# A Risk Prediction Model for Evaluating the Disease Progression of COVID-19 Pneumonia

**DOI:** 10.3389/fmed.2020.556886

**Published:** 2020-11-05

**Authors:** Guodong Cao, Pengping Li, Yuanyuan Chen, Kun Fang, Bo Chen, Shuyue Wang, Xudong Feng, Zhenyu Wang, Maoming Xiong, Ruiying Zheng, Mengzhe Guo, Qiang Sun

**Affiliations:** ^1^School of Medicine, Zhejiang University, Hangzhou, China; ^2^The First Affiliated Hospital of Anhui Medical University, Hefei, China; ^3^The First People's Hospital of Xiaoshan District, Hangzhou, China; ^4^Department of Radiation and Medical Oncology, Zhongnan Hospital of Wuhan University, Wuhan University, Wuhan, China; ^5^Hubei Key Laboratory of Tumor Biological Behaviors, Wuhan University, Wuhan, China; ^6^Hubei Cancer Clinical Study Center, Wuhan, China; ^7^Yinchuan Maternal and Child Health Hospital, Yinchuan, China; ^8^The Fourth People's Hospital of Ningxia Hui Autonomous Region, Yinchuan, China; ^9^Department of Infectious Disease, Zhongnan Hospital of Wuhan University, Wuhan, China; ^10^Jiangsu Key Laboratory of Biological Cancer, Cancer Institute, Xuzhou Medical University, Xuzhou, China

**Keywords:** sSARS—CoV-2, meta-analysis, COVID 19, risk prediction model, disease progression

## Abstract

**Background and Objective:** The epidemic of coronavirus disease 2019 (COVID-19) pneumonia caused by infection with severe acute respiratory syndrome coronavirus 2 (SARS-CoV2) has expanded from China throughout the world. This study aims to estimate the risk of disease progression of patients who have been confirmed with COVID-19.

**Methods:** Meta-analysis was performed in existing literatures to identify risk factors associated with COVID-19 pneumonia progression. Patients with COVID-19 pneumonia were admitted to hospitals in Wuhan or Hangzhou were retrospectively enrolled. The risk prediction model and nomogram were developed from Wuhan cohort through logistic regression algorithm, and then validated in Hangzhou and Yinchuan cohorts.

**Results:** A total of 270 patients admitted to hospital between Dec 30, 2019, and Mar 30, 2020, were retrospectively enrolled (Table 1). The development cohort (Wuhan cohort) included 87 (43%) men and 115 (57%) women, and the median age was 53 years old. Hangzhou validation cohort included 20 (48%) men and 22 (52%) women, and the median age was 59 years old. Yinchuan validation cohort included 12 (46%) men and 14 (54%) women, and the median age was 44 years old. The meta-analysis along with univariate logistic analysis in development cohort have shown that age, fever, diabetes, hypertension, CREA, BUN, CK, LDH, and neutrophil count were significantly associated with disease progression of COVID-19 pneumonia. The model and nomogram derived from development cohort show good performance in both development and validation cohorts.

**Conclusion:** The severe COVID-19 pneumonia is associated with various types of risk factors including age, fever, comorbidities, and some laboratory examination indexes. The model integrated with these factors can help to evaluate the disease progression of COVID-19 pneumonia.

## Introduction

Since December, 2019, China reported a cluster of cases of pneumonia with unknown cause in Wuhan, Hubei (1). On Jan 7, 2020, Chinese health authorities have confirmed these cases were associated with a novel coronavirus, severe acute respiratory syndrome corona virus 2 (SARS-CoV2; previously called 2019-nCoV) via next generation sequencing analysis of patients' respiratory tract samples (2, 3). SARS-CoV2 is the seventh member of Coronaviridae, which has been shown to infect human cells through interacting with the angiotensin-converting enzyme 2 (ACE2) receptor on the cell surface (4). ACE2 receptor is wildly distributed in various types of human cells including type II alveolar cells, renal tubular cells, Leydig cells and so on (5). Thus, SARSCoV2 possesses a strong ability to infect humans.

Most of the original cases of coronavirus disease 2019 (COVID-19) pneumonia were reported to have been exposed to the Huanan seafood market in Wuhan (6). However, the medical and nursing staffs, patients without exposure to the market but with a history of travel to Wuhan have been found to be infected by SARS-CoV2, suggesting that human-to-human transmission is occurring (7, 8). The number of diagnosed cases has been increasing rapidly: by March 27 2020, more than 500,000 cases of COVID-19 pneumonia had been reported in China and other countries worldwide (including Japan, South Korea, Spain, Italy, the UK, and the USA), and over 23,000 patients had died, equivalent to a case fatality rate of around 4% (9). Epidemic prevention is becoming increasingly severe.

Similar as respiratory diseases caused by other beta-coronaviruses such as severe acute respiratory syndrome (SARS) and Middle East respiratory syndrome (MERS) (10, 11). Patients with both mild and severe COVID-19 pneumonia showed fever, dry cough, and dyspnea symptoms (6). Furthermore, patients with severe COVID-19 pneumonia were more likely to progress to acute respiratory distress syndrome (ARDS), which had relatively higher case fatality rate (12). However, little studies were reported about evaluating the risk of disease progression of COVID-19 pneumonia.

In this study, we employed a meta-analysis of 6,061 cases of COVID-19 from 32 studies. The results showed that severe COVID-19 pneumonia was obviously correlated with severe complications, including ARDS, shock, acute kidney injury and acute cardiac injury. And the comorbidities significantly increased the risk of progressive COVID-19. In addition, a total of 270 COVID-19 pneumonia patients were collected from Wuhan, Hangzhou, and Yinchuan. A predictive model and nomogram were then established based on previously identified risk factors including age, fever, comorbidities, CK, LDH, CREA, BUN, and neutrophil count to predict the risk of disease progression in Wuhan cohort. A nomogram is a statistical instrument that accounts for numerous variables to predict an outcome of interest (13). The nomogram showed great performance in predicting the probability of severe COVID-19 pneumonia, which was further validated in two independent cohorts.

## Materials and Methods

### Meta-Analyses

Thirty-two eligible studies were analyzed by performing meta-analysis. OR, RR, SMD, 95% CI as well as forest plots were calculated via stata 12.0. Specific analysis procedures including search strategy, inclusive and exclusive criteria, data extraction, quality evolution and statistics were provided in Supplementary Texts 1, 2.

### Study Design and Participants

This was a retrospective study done at two centers in Wuhan and Hangzhou. Patients with confirmed COVID-19 pneumonia were admitted to Zhongnan Hospital of Wuhan University or the First People's Hospital of Xiaoshan District or The Fourth People's Hospital of Ningxia Hui Autonomous Region were retrospectively enrolled (Table 1).

**Table 1:** Demographic and clinical characteristics.

**Parameters**	**Development cohort (*n* = 202)**	**Hangzhou validation cohort (*n* = 42)**	**Yinchuan validation cohort (*n* = 26)**
**CLINICAL CHARACTERISTICS**			
Age (median, range, y)	53 (20–88)	59 (20–87)	44 (15–70)
**Gender**			
Male	87 (43.1%)	20 (47.6%)	12 (46.2%)
Female	115 (56.9%)	22 (52.4%)	14 (53.8%)
**Status**			
Mild	102 (55.1%)	26 (61.9%)	20 (76.9%)
Severe	83 (44.9%)	16 (38.1%)	6 (23.1%)
**Fever**			
Yes	145 (72.1%)	30 (71.4%)	19 (73.1%)
No	56 (27.9%)	12 (28.6%)	7 (26.9%)
**COMORBIDITIES**			
**Hypertension**			
Yes	31 (15.9%)	7 (16.7%)	4 (15.4%)
No	164 (84.1%)	35 (83.3%)	22 (84.6%)
**Diabetes**			
Yes	17 (8.7%)	5 (11.9%)	0 (0%)
No	178 (91.3%)	37 (88.1%)	26 (100%)
**LABORATORY INDICTORS**			
BUN (mmol/L)	4.7 ± 2.3	5.7 ± 4.4	3.9 ± 1.1
CREA (μmol/L)	71.5 ± 26.2	80.6 ± 31.0	65.8 ± 18.2
CK (U/L)	171.1 ± 441.3	97.6 ± 86.0	56.9 ± 26.8
LDH (U/L)	284.2 ± 207.4	252.4 ± 181.5	241.0 ± 55.3
Neutrophil (10^9^/L)	3.9 ± 3.7	5.0 ± 5.7	3.2 ± 1.5

Next-generation sequencing or real-time PCR of throat swab specimens were used to confirm the SARS-CoV2 infection of each patient according to a previously published protocol (14). The following primers or probe targeted to the envelope gene of SARS-CoV2 were used: Forward primer: 5′-GACCCCAAAATCAGCGAAAT-3′; Reverse primer: 5′-TCTGGTTACTGCCAGTTGAATCTG-3′; Probe: 5′-FAM-ACCCCGCATTACGTTTGGTGGACC-BHQ1-3′.

Patients with respiratory rate over 30 per min, or SpO_2_ <93%, or PaO_2_/FiO_2_ <300 mmHg were considered as severe cases (15). The clinical characteristics, laboratory findings, and comorbidities of the patients were recorded at the time of admission to hospital. The study is approved by the Ethics Committee of the Zhongnan Hospital of Wuhan University, the First hospital of Xiaoshan District and The Fourth People's Hospital of Ningxia Hui Autonomous Region under the accession 2020035.

### Statistical Analysis

Normality of the data was evaluated using the Kolmogorov-Smirnov test. Data with normal distributions were presented as mean ± SD, data with non-normally distribution were presented as median (IQR), and categorical variables as frequency (%). Differences between two groups were analyzed by Fisher's exact test (for categorical variables) or Mann-Whitney *U-*test (for continuous variables). The hazard ratios (HRs) and corresponding 95% CIs were calculated using univariate or multivariate logistic regression algorithm.

### Model Development and Valudations

For the development of the nomogram, we tested the significance of potential risk factors by univariate logistic regression algorithm and further filtered by meta-analysis. As a result, eight important predictors of risk of severe COVID-19 pneumonia were to create the model via multivariate logistic regression. The models produce a linear predictor, which is represented as a patient's predicted log hazard. We tested the accuracy of the model as well as nomogram derived from the development cohort in the validation cohort by discrimination, ROC (receiver operating characteristic curve) analysis and calibration curve. The nomogram converts the risk of each variable into a points system, which can be added to produce an overall risk estimate.

The discrimination is evaluated by the c-statistic, which measures the capability of the model to predict a high risk for a patient who is considered as high risk. The closer that c-statistic is to one, the better the discrimination, and a value of 0.5 indicates that the model is not better than chance. The calibration curve of the model measures the relationship between the outcomes predicted by the model and the actual outcomes in indicated cohort. A 45° line indicates perfect calibration, which the predicted outcome of the model perfectly matches the patient's observed outcome. Any deviation above or below the 45° line indicates underprediction or overprediction, respectively. The ROC analysis is evaluated by AUC (area under the curve), which measures the sensitivity and specificity of the model. The closer that AUC is to one, the more sensitive and specific the model. All the statistics were performed using R project (version 3.4.4).

## Results

**Figure 1:**
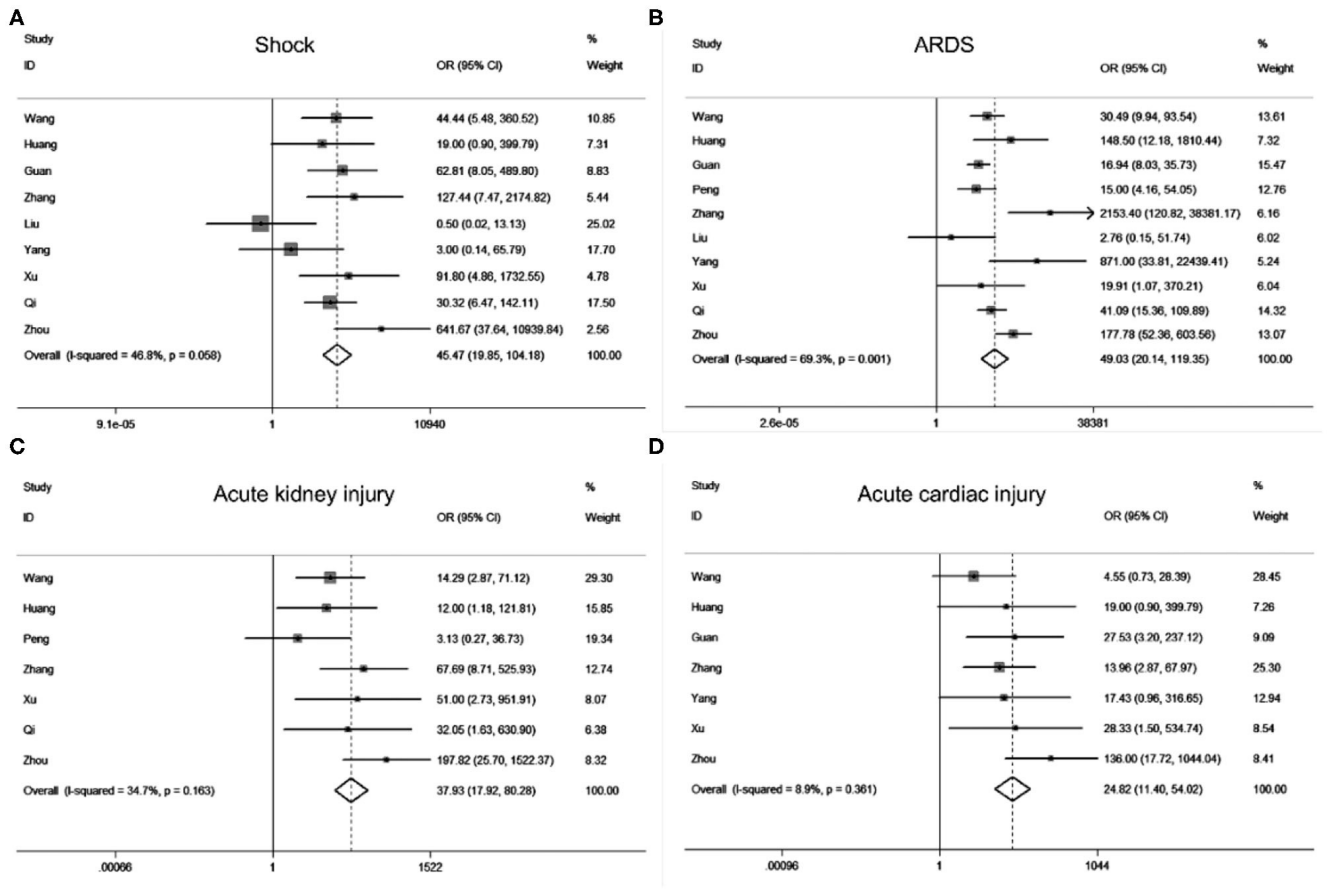
Forest plots of odds ratios (OR) showed the associations between complications including shock **(A)**, ARDS **(B)**, acute kidney injury **(C)**, as well as acute cardiac injury **(D)** and disease progression of COVID-19 pneumonia.

According to our search strategy, a total of 142 studies were collected from the online database. Filtered by the criteria of inclusion and exclusion, we finally retained 32 (Supplementary Figure 1) studies for further meta-analysis, and no publication bias exists (Supplementary Figure 2). Meta-analysis results revealed that acute cardiac injury [odds ratio [OR] = 37.93, 95% confidence interval [CI]: 17.92–80.28; *P* < 0.001], acute kidney injury [OR = 24.82, 95% CI: 11.40–54.02; *P* < 0.001), ARDS (OR = 49.03, 95% CI: 20.14–119.35; *P* < 0.001), shock (OR = 45.48, 95% CI: 19.85–104.18; *P* < 0.001) occurred frequently in patients with severe COVID-19 pneumonia compared with the ordinary cases (Figure 1). The death event [risk ratio [RR] = 30.09, 95% CI: 11.46–79.01; *P* < 0.001 was more common in disease progression in COVID-19 pneumonia. The detailed meta-analysis results were summarized in Supplementary Table 1.

**Figure 2:**
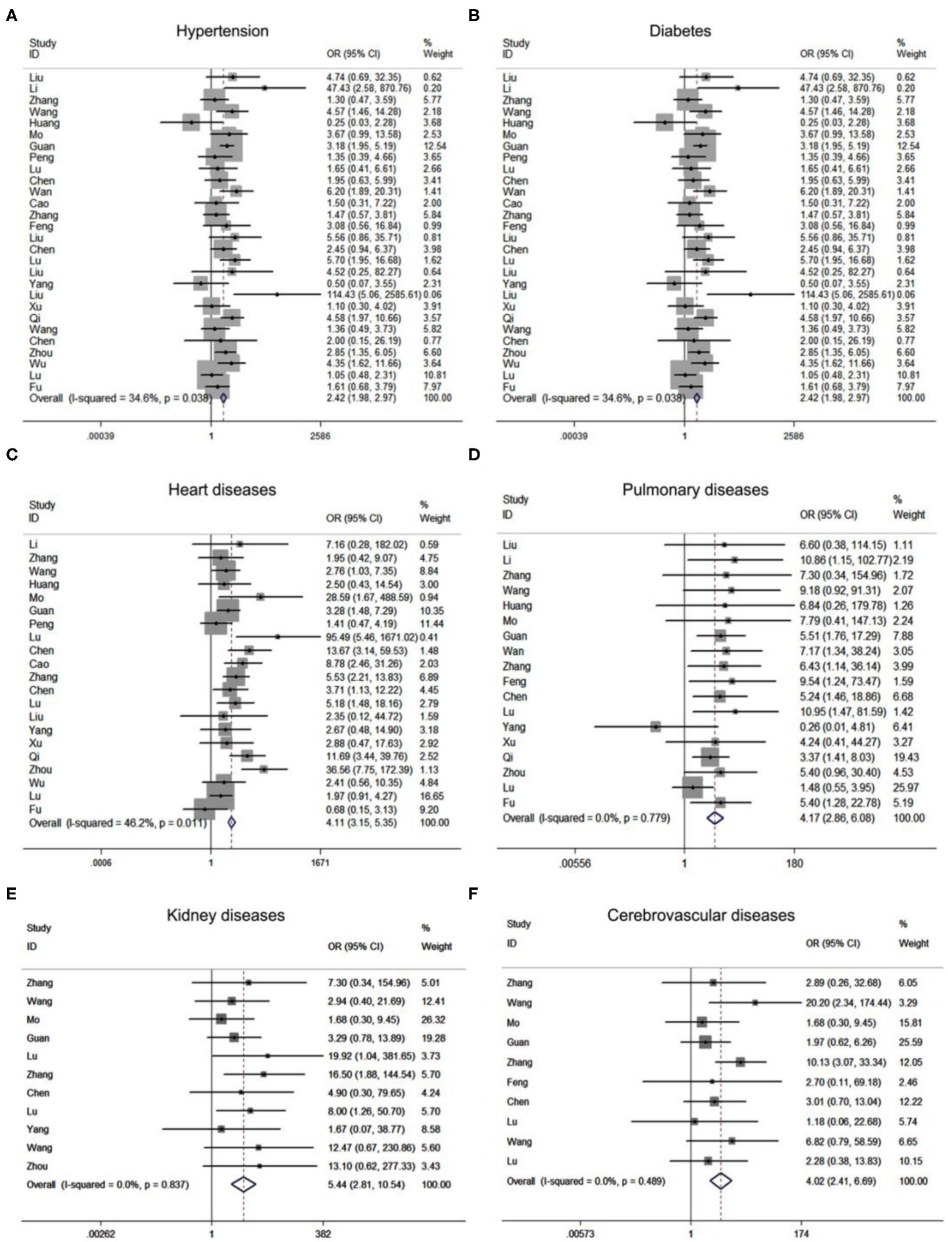
Forest plots of odds ratios (OR) showed the associations between comorbidities including hypertension **(A)**, diabetes **(B)**, cardiac diseases **(C)**, pulmonary diseases **(D)**, renal diseases **(E)**, as well as cerebrovascular diseases **(F)**, and disease progression of COVID-19 pneumonia.

To investigate the variables associated with severe COVID-19 pneumonia, we analyzed the relationship between patients' clinical characteristics and severe COVID-19 pneumonia risk through meta-analysis. Supplementary Table 2 showed that age [standardized mean difference [SMD] = 2.04, 95% CI: 1.45–2.63, *P* < 0.001] and gender [OR = 1.57, 95% CI: 1.36–1.80, *P* < 0.001] held different distribution in severe and mild COVID-19 pneumonia groups. Additionally, patients with comorbidities, including hypertension [OR = 2.53, 95% CI: 1.89–3.37, *P* < 0.001), diabetes (OR = 2.43, 95% CI: 1.98–2.97, *P* < 0.001), cancer (OR = 1.73, 95% CI: 1.07–2.78, *P* = 0.025), cerebrovascular (OR = 4.02, 95% CI: 2.41–6.60, *P* < 0.001), cardiac disease (OR = 4.11, 95% CI: 3.15–5.35, *P* < 0.001), renal disease (OR = 5.44, 95% CI: 2.81–10.54, P < 0.001), and pulmonary disease (OR = 4.17, 95% CI: 2.86–6.08, *P* < 0.001) may be closely related with the risk of patients infected with SARS-CoV2 progressed to severe COVID-19 pneumonia (Figure 2). In addition, different clinical symptoms including cough, fatigue, fever, muscular soreness, and CT performance were also correlated with severe COVID-19 pneumonia. The above symptoms may frequently occur in patients with progressive COVID-19 pneumonia. Next, we analyzed the relationship between patients' laboratory findings and severe COVID-19 pneumonia risk. The results showed that hepatic function indexes albumin, ALT, AST, renal function indexes BUN, CREA, cardiac function indexes CK, CK-MB, LDH, cTnI, MYO, coagulation function indexes D-dimer, PLT, PT, blood routine indexes Hb, lymphocyte, neutrophil, CD4 lymphocyte, CD8 lymphocyte, inflammation indexes WBC, CRP, PCT, ESR, ferritin, and electrolyte indexes Na^+^ were obviously changed in patients with severe COVID-19 pneumonia compared with mild COVID-19 pneumonia patients. Moreover, the Supplementary Table 3 showed that blood routine indexes Hb, lymphocyte (SMD = −2.35, 95% CI: −2.85–1.86; Supplementary Figure 3A), WBC (SMD = 1.35, 95% CI: 0.49–2.22; Supplementary Figure 3B), neutrophil (SMD = 2.21, 95% CI: 1.70–2.73; Supplementary Figure 3C), CD4 lymphocyte, CD8 lymphocyte, inflammation indexes CRP (SMD = 4.28, 95% CI: 3.23–5.33; Supplementary Figure 3D), PCT, ESR, and ferritin were obviously changed, which indicated that the severe patients may suffer from inflammatory factor storm. At the same time hepatic function indexes ALT (SMD = 1.29, 95% CI: 0.84–1.74; Supplementary Figure 4A) and AST (SMD = 2.01, 95% CI: 1.20–2.81; Supplementary Figure 4B), renal function indexes CREA (SMD = 1.20, 95% CI: 0.72–1.68; Supplementary Figure 4C) and BUN (SMD = 2.44, 95% CI: 1.52–3.36; Supplementary Figure 4D), cardiac function indexes CK (SMD = 4.04, 95% CI: 2.73–5.36; Supplementary Figure 4E), LDH (SMD = 1.64, 95% CI: 0.71–2.56; Supplementary Figure 4F), coagulation function indexes D-dimer, PLT, PT, and electrolyte indexes Na^+^ were increased in patients with severe COVID-19 pneumonia compared with mild COVID-19 pneumonia patients, which indicated patients with severe COVID-19 pneumonia may be suffering from multi-organ dysfunctions.

Together, the meta-analysis results revealed that age (HR = 1.31, 95% CI: 1.17–1.47; *P* < 0.001), diabetes (HR = 1.71, 95% CI: 1.16–2.54; *P* = 0.007), heart diseases (HR = 5.02, 95% CI: 1.47–17.10; *P* = 0.01), hypertension (HR = 2.11, 95% CI: 1.60–2.79; *P* < 0.001), pulmonary diseases (HR = 3.60, 95% CI: 1.91–6.78; *P* < 0.001), blood oxygen saturation (HR = 0.94, 95% CI: 0.89–0.99; *P* = 0.025), CREA (HR = 1.08, 95% CI: 1.02–1.15; *P* = 0.007), BUN (HR = 1.54, 95% CI: 1.28–1.87; *P* < 0.001), neutrophil (HR = 1.17, 95% CI: 1.00–1.38; *P* = 0.050), PCT (HR = 4.07, 95% CI: 1.05–15.73; *P* = 0.042), serum ferritin (HR = 4.59, 95% CI: 2.00–10.55; *P* < 0.001), CK (HR = 2.13, 95% CI: 1.44–3.15; *P* < 0.001), LDH (HR = 2.15, 95% CI: 1.32–3.51; *P* = 0.002) and cTnI (HR = 4.87, 95% CI: 1.48–16.01; *P* = 0.009) were potential risk factors for disease progression of COVID-19 pneumonia (Supplementary Table 4).

To further validate the potential risk factors for COVID-19 pneumonia progression, we collected 202 COVID-19 pneumonia cases from Zhongnan Hospital of Wuhan University (termed as Wuhan cohort). The median age of Wuhan cohort is 53 years [Interquartile Range [IQR], 20–88]. About 20% of all patients have comorbidities such as hypertension (15.3%) and diabetes (8.4%) (Table 1). Similarly, patients with severe COVID-19 pneumonia are elder and with different comorbidities including hypertension and diabetes (Table 2). We further analyzed the relationship between potential risk factors identified by meta-analysis using the univariate logistic regression (Supplementary Figure 5).

**Table 2:** Clinical characteristic of 202 COVID−19 pneumonia patients from Zhongnan Hospital of Wuhan University.

**Parameters**	**Total cases (*N* = 202)**	**Mild cases (*N* = 102)**	**Severe cases (*N* = 83)**	***P-*value**	**Normal range**
**CLINICAL CHARACTERISTICS**					
Age (median, range, y)	53 (20–88)	46 (20–83)	62 (23–88)	<0.001	
**Gender**				0.038	
Male	87 (43.1%)	36 (35.3%)	42 (50.6%)		
Female	115 (56.9%)	66 (64.7%)	41 (49.4%)		
**Fever**				0.021	
Yes	145 (72.1%)	66 (64.7%)	66 (80.5%)		
No	56 (27.9%)	36 (35.3%)	16 (19.5%)		
**Cough**				0.076	
Yes	106 (52.7%)	47 (46.1%)	49 (59.8%)		
No	95 (47.3%)	55 (53.9%)	33 (40.2%)		
**COMORBIDITIES**					
**Hypertension**				<0.001	
Yes	31 (15.9%)	7 (6.9%)	24 (31.6%)		
No	164 (84.1%)	95 (93.1%)	52 (68.4%)		
**Diabetes**				0.002	
Yes	17 (8.7%)	3 (2.9%)	14 (18.4%)		
No	178 (91.3%)	99 (97.1%)	62 (81.6%)		
**LABORATORY INDICTORS**					
**Hepatic Function**					
ALT (U/L)	35.6 ± 37.0	24.9 ± 23.0	46.8 ± 44.1	<0.001	7–45
AST (U/L)	42.6 ± 53.5	26.0 ± 12.7	60.0 ± 73.5	<0.001	13–35
Total bilirubin (μmol/L)	12.7 ± 10.5	11.1 ± 4.8	13.7 ± 7.8	0.029	5–21
Albumin(g/L)	37.7 ± 5.0	40.0 ± 3.5	35.0 ± 5.2	<0.001	40–55
**Renal Function**					
BUN (mmol/L)	4.7 ± 2.3	4.1 ± 1.6	5.6 ± 2.9	<0.001	2.8–7.6
CREA (μmol/L)	71.5 ± 26.2	65.3 ± 21.5	78.1 ± 29.2	<0.001	49–90
**Cardiac Function**					
CK (U/L)	171.1 ± 441.3	92.8 ± 95.5	238.8 ± 546.2	<0.001	<145
CK–MB (U/L)	18.0 ± 23.9	14.2 ± 22.6	19.1 ± 18.2	<0.001	0–25
LDH (U/L)	284.2 ± 207.4	192.8 ± 93.3	392.6 ± 254.2	<0.001	125–243
Myoglobin (U/L)	215.0 ± 371.6	31.0 ± 22.4	248.7 ± 413.0	0.040	<140.1
NT–proBNP (pg/mL)	542.7 ± 877.1	200.2 ± 326.3	671.5 ± 1000.5	0.313	0–900
**Coagulation Function**					
PT (s)	13.0 ± 1.6	12.6 ± 1.1	13.4 ± 2.0	0.008	9.4–12.5
APTT (s)	31.2 ± 3.2	31.3 ± 2.9	31.0 ± 3.7	0.163	25.1–36.5
D–dimer (ng/L)	1416.2 ± 5396.9	292.2 ± 453.9	3126.9 ± 8432.6	<0.001	0–500
Fibrinogen (mg/L)	410.0 ± 111.7	398.9 ± 105.1	418.3 ± 117.4	0.443	238–498
PLT (10^12^/L)	185.5 ± 68.0	191.6 ± 61.9	178.4 ± 74.6	0.059	125–350
**Inflammatory Index**					
WBC (10^9^/L)	5.3 ± 3.0	4.4 ± 1.8	6.4 ± 3.9	0.002	3.5–9.5
Monocyte (10^9^/L)	0.5 ± 0.9	0.4 ± 0.2	0.6 ± 1.4	0.968	0.1–0.6
Neutrophil (10^9^/L)	3.9 ± 3.7	2.8 ± 1.6	5.3 ± 5.1	<0.001	1.8–6.3
Eosinophil (10^9^/L)	0.04 ± 0.17	0.07 ± 0.23	0.02 ± 0.05	0.008	0.02–0.52
CRP (mg/L)	39.8 ± 61.3	17.5 ± 21.7	77.9 ± 84.7	<0.001	0–10
**Electrolyte**					
K^+^ (mmol/L)	4.03 ± 0.65	4.05 ± 0.67	3.99 ± 0.66	0.566	3.5–5.3
Na^+^ (mmol/L)	138.4 ± 5.9	140.1 ± 6.2	136.9 ± 4.0	<0.001	137–147
Ca^+^ (mmol/L)	2.16 ± 0.17	2.22 ± 0.12	2.07 ± 0.21	<0.001	2.11–2.52
**Survival Status**				0.001	
Alive	190 (94.1%)	95 (99.0%)	71 (86.6%)		
Death	12 (5.9%)	1 (1.0%)	11 (13.4%)		

The results showed that age (HR = 1.07, 95% CI: 1.05–1.09; *P* < 0.001), fever (HR = 2.25, 95% CI: 1.28–4.04; *P* < 0.001), diabetes (HR = 7.45, 95% CI: 2.76–25.26; *P* = 0.002), hypertension (HR = 6.26, 95% CI: 3.02–14.07; *P* < 0.001), BUN (HR = 1.39, 95% CI: 1.21–1.63; *P* < 0.001), CREA (HR = 1.02, 95% CI: 1.01–1.04; *P* = 0.002), CK (HR = 1.00, 95% CI: 1.00–1.01; *P* = 0.03), LDH (HR = 1.01, 95% CI: 1.00–1.02; *P* < 0.001), and neutrophil (HR = 1.43, 95% CI: 1.25–1.68; *P* < 0.001) remained significantly associated with the disease progression of COVID-19 pneumonia in both logistic and meta analyses (Table 3).

**Table 3:** Univariate logistic results of different variables in development cohort.

**Parameters**	**HR**	**95%CI**	**Z-value**	***P-*value**
Age	1.07	1.05–1.09	5.56	<0.001
Gender (Male vs. Female)	1.88	1.15–3.10	2.09	0.04
Fever (Yes vs. No)	2.25	1.28–4.04	2.34	0.02
Cough (Yes vs. No)	1.85	1.13–3.06	2.03	0.04
Hypertension (Yes vs. No)	6.26	3.02–14.07	3.96	<0.001
Diabetes (Yes vs. No)	7.45	2.76–25.26	3.06	0.002
ALT	1.03	1.02–1.05	3.53	<0.001
AST	1.06	1.04–1.08	4.87	<0.001
Total bilirubin	1.07	1.03–1.12	2.54	0.01
Albumin	0.75	0.69–0.81	−5.79	<0.001
BUN	1.39	1.21–1.63	3.71	<0.001
CREA	1.02	1.01–1.04	3.08	0.002
CK	1.00	1.00–1.01	2.14	0.03
CK–MB	1.01	1.00–1.03	1.37	0.17
LDH	1.01	1.01–1.02	5.06	<0.001
Myoglobin	1.09	1.02–1.22	1.73	0.08
NT–proBNP	1.00	1.00–1.01	0.64	0.52
PT	1.42	1.17–1.77	2.80	0.01
APTT	0.97	0.89–1.06	−0.53	0.59
D–dimer	1.00	1.00–1.00	2.77	0.01
Fibrinogen	1.00	1.00–1.00	1.02	0.31
PLT	1.00	0.99–1.00	−1.26	0.21
WBC	1.30	1.16–1.47	3.69	<0.001
Monocyte	1.48	0.95–3.91	0.79	0.42
Neutrophil	1.43	1.25–1.68	4.02	<0.001
Eosinophil	0.02	0.00–0.65	−1.38	0.17
CRP	1.03	1.02–1.04	3.84	<0.001
K^+^	0.86	0.54–1.32	−0.55	0.58
Na^+^	0.81	0.74–0.89	−3.75	<0.001
Ca^+^	0.0004	0.000012–0.0038	−4.76	<0.001

**Figure 3:**
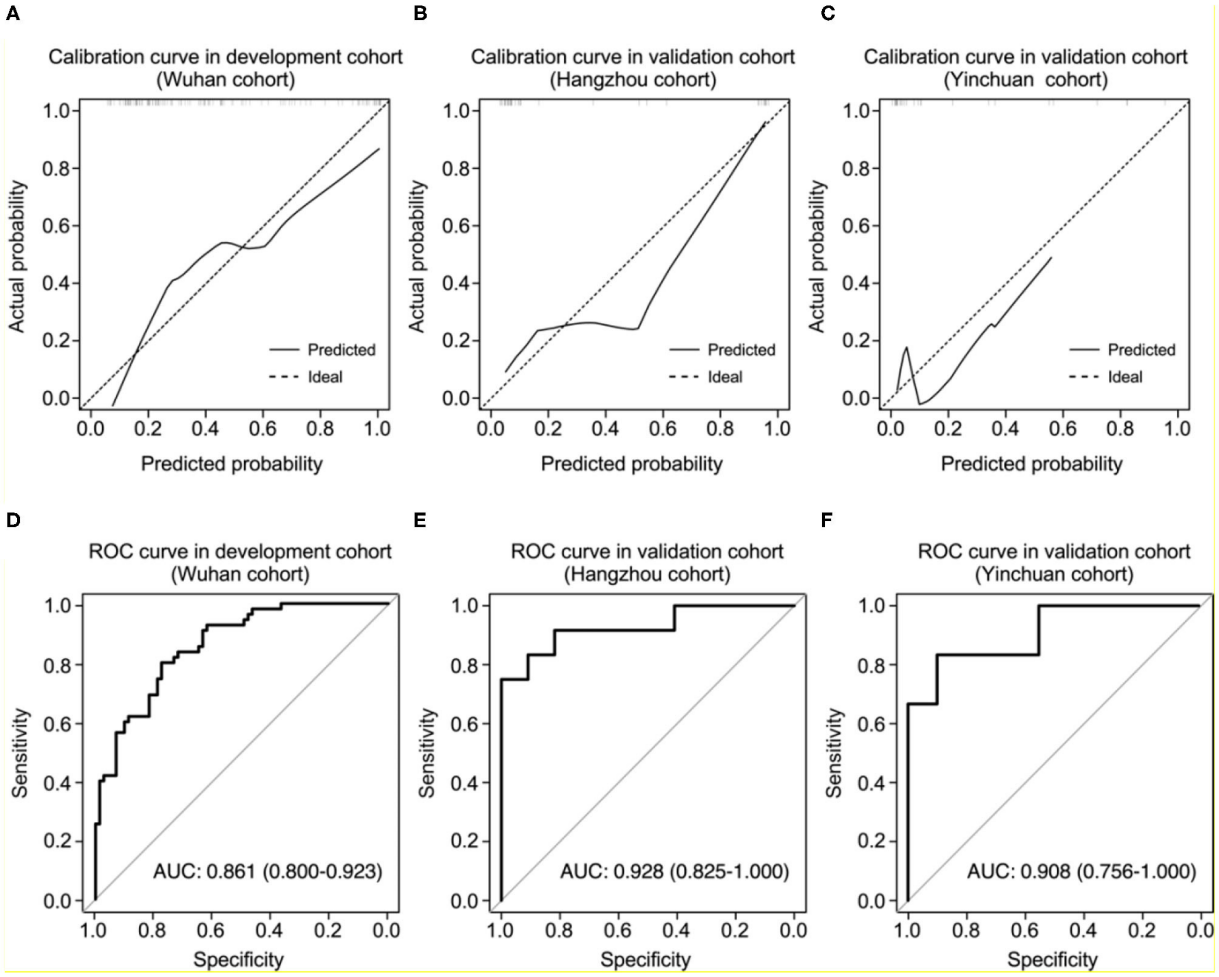
Performance of the nomogram in development and validation cohorts. **(A–C)** Calibration curves of the nomogram for predicting of the probability of severe COVID-19 pneumonia in development **(A)**, Hangzhou validation **(B)**, and Yinchuan validation cohorts **(C)**. The histogram at the top of the plot shows the distribution of the predicted probabilities. **(D–F)** Receiver operating characteristic curves of sensitivity and specificity of the nomogram for predicting severe COVID-19 pneumonia in development **(D)**, Hangzhou validation **(E)**, and Yinchuan validation cohorts **(F)**.

To evaluate the probability of the patient with severe COVID-19 pneumonia, we sought to develop a model for predicting the risk of disease progression using multivariate logistic regression algorithm with the above variables in Wuhan cohort. The final model contained nine investigated variables (including age, fever, hypertension, diabetes, BUN, CREA, CK, LDH, and neutrophil count) and one interaction term. The model for predicting the risk of severe pneumonia had a c-statistic of 0.86, with good calibration curve (Figure 3). The ROC analysis in Wuhan cohort revealed that the model had high sensitivity and specificity (Figure 3). Moreover, we collected another two independent cohorts of patients from Hangzhou and Yinchuan as validation cohorts to evaluate the performance of the model. The Hangzhou validation cohort consisted of 42 patients. 16 (38%) patients were diagnosed as severe COVID-19 pneumonia, and 26 patients were diagnosed as mild COVID-19 pneumonia (Table 1). The model for predicting the risk of severe pneumonia had a c-statistic of 0.93, with great calibration curve in validation cohort (Figure 3). The model had an AUC of 0.928, which indicated it also had good performance in validation cohort (Figure 3). The Yinchuan validation cohort consisted of 26 patients. 6 (23%) patients were diagnosed as severe COVID-19 pneumonia (Table 1). The model for predicting the risk of severe pneumonia had a c-statistic of 0.90, with great calibration curve in validation cohort (Figure 3). The model had an AUC of 0.908, which indicated it also had good performance in validation cohort (Figure 3).

To prevent the overfitting of our model, we performed correlation analysis between the biological test parameters used in our model, the results showed that the CREA level was significantly correlated with the BUN level in COVID-19 patients (*R* = 0.77, *P* < 0.001; Supplementary Figure 6). We sought to interact these two parameters in our model, through the AUC of model increased to 0.874 in the development cohort (Supplementary Figure 6), the AUC dropped to 0.837 and 0.900 in Hangzhou and Yinchuan validation cohort, respectively (Supplementary Figure 6). Therefore, we use all the variables to generate the model without interactions.

**Figure 4:**
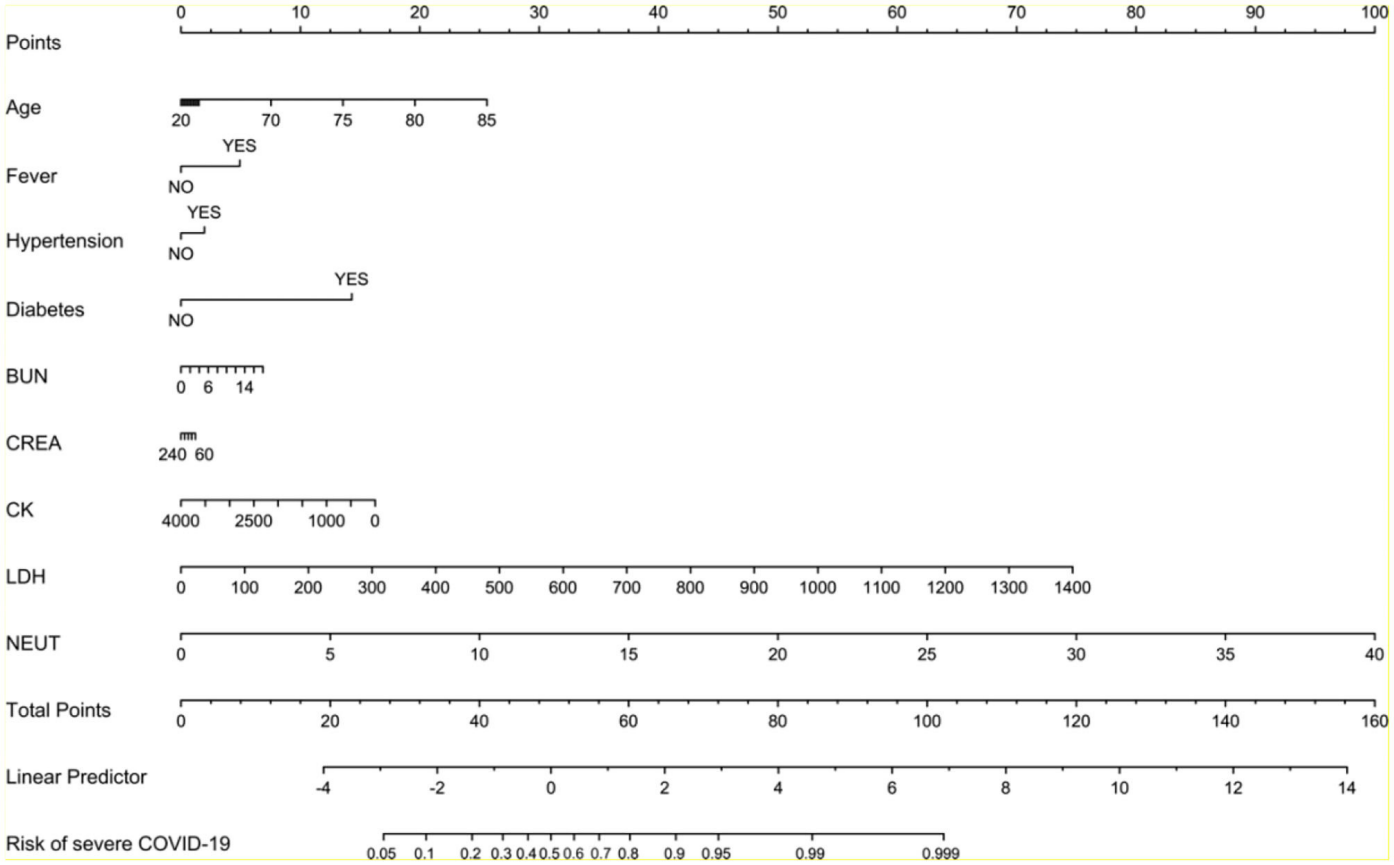
Nomogram to predict chance of being severe COVID-19 pneumonia.

We created the nomogram to predict the probability of severe COVID-19 pneumonia (Figure 4). The line labeled Points was used to calculate the points referred to each of the nine risk factors. The subsequent lines (lines 2–10 in Figure 4) are the risk factors involved in this model. The value for each variable is located on these lines and a vertical line is drawn up to the points line to find the points associated with each value. All of the points are summed and the total located on the Total points line. According to the total points, we could get the COVID-19 pneumonia of severe COVID-19 pneumonia for each patient.

## Discussion

The Coronaviridae is a family of enveloped, non-segmented, positive-stranded RNA viruses, which are widely distributed in humans and other mammals such as bats, masked palm civets, and pangolins (16, 17). Six of these coronaviruses are found to infect humans previously, and four of them only mild respiratory symptoms similar to the common cold. However, MERS-CoV and SARS-CoV, two kinds of beta-coronaviruses, infected more than 1,000 and 8,000 patients with high case fatality rates (37% for MERS-CoV and 10% for SARS-CoV), respectively (18, 19). The novel coronavirus −19, which is also considered as SARS-CoV2, is the seventh member of the Coronaviridae family found to infect human beings (20). Though the case fatality rate of SARS-CoV2 so far is lower than that of SARS-CoV or MERS-Cov diseases, SARS-CoV2 is contagious in humans and is the cause of the ongoing pandemic that has been designated a Public Health Emergency of International Concern by the World Health Organization (WHO) (21).

In our study, we employed a meta-analysis to identify relevant variables for promoting COVID-19 pneumonia progression. Severe COVID-19 pneumonia was obviously correlated with severe complications including ARDS (OR = 49.03), shock (OR = 45.48), acute kidney injury (OR = 24.82), acute cardiac injury (OR = 37.93), progressive COVID-19 also enhanced the death risk (RR = 30.09). Previous studies have implied the SARS-CoV2 infection may cause multi-organ injuries, our results further supported their conclusion by making evidence-based medicine research (22, 23). Moreover, Age and comorbidities such as hypertension, diabetes, cancers, heart diseases, pulmonary diseases, kidney diseases as well as cerebrovascular diseases can significantly increase the risk of COVID-19 progression. Meanwhile, we also found that clinical symptoms might not contribute to the disease progression of COVID-19 pneumonia. Laboratory findings especially the renal function index (CREA, BUN), myocardial enzymonram (CK, LDH), and neutrophil counts in severe COVID-19 patients were sustained higher than that of the ordinary patients.

Age and comorbidities were important potential risk factors in progressive COVID-19 pneumonia (24). Elder patients could progress to COVID-19 pneumonia due to the hypo-immunity and comorbidities. Laboratory examinations could reflect the different statuses of organs. We observed that a range of indicators representing functions of different organs had changed in COVID-19 patients, especially in severe COVID-19 patients via meta-analysis, suggesting the existence of multi-organ dysfunctions in patients with severe COVID-19 pneumonia. Organ injuries and progressive COVID-19 pneumonia may be reciprocal causation, progressive COVID-19 caused organ injuries, and indexes of organ injuries could in turn help us to monitor the risk of COVID-19 pneumonia progression. The decision to evaluate disease progression COVID-19 patients based on solely index might be misleading. However, there are no explicit standards or risk models to assist diagnosis. Thus, we collected a total of 270 COVID-19 patients to establish a model for evaluating the risk of COVID-19 pneumonia progression.

In our development cohort, concentrations of C-reactive protein were increased in most patients, similar as observed in previous beta-coronavirus infections (10, 11). In the subgroup of patients with severe COVID-19 pneumonia, concentrations of C-reactive protein (74.3 mg/L) were higher than those in mild patients. The concentration of albumin was significantly reduced in severe group patients, which might lead to albuminemia. This finding is similar to those of a previous study of patients with H1N1 infection (25). Furthermore, renal function index (CREA and BUN), myocardial enzymonram (CK and LDH), and liver function index (ALT and AST) were elevated in patients with severe COVID-19 pneumonia. Previous studies also showed that the severe COVID-19 pneumonia could cause kidney dysfunctions based on biopsy samples (26). Thus, our findings indicate that patients with severe COVID-19 pneumonia may be suffered with multiple organ dysfunctions.

Several variables have been documented to be associated with severe COVID-19 pneumonia, such as cancer, hypertension, heart diseases and so on (24). However, no study integrates these risk factors to predict the probability of severe COVID-19 pneumonia. First, we screened the predictive variables for severe COVID-19 pneumonia using the 202 patients from Wuhan cohort. Eight risk factors were found to be significantly correlated with severe COVID-19 pneumonia, and we subsequently created a model and a nomogram based on these risk factors by multivariate logistic regression algorithm. To further validate the validity of the model and nomogram, we collected another two validation cohorts of COVID-19 pneumonia patients from Hangzhou and Yinchuan, the calibration, discrimination and ROC analysis together reveal that the model we built has good performance in two independent validation cohorts (Figure 3).

Our prediction model and nomogram can help doctors to evaluate the risk of disease progression of COVID-19 pneumonia patients on admission to hospital. The nomogram summed points showed the risk of each patient, sensitively reflecting the dysfunctions of multi-organs and the risk score might be related to the outcome of the patient. Accurate risk evaluation may also assist therapeutic decision-making for different patients. According to previous report, steroid drugs may bring no benefits for patients with mild COVID-19 pneumonia, indicating that patients with low risk may not accept steroid drugs treatment (27, 28). In addition, the decision to make disease managements for COVID-19 pneumonia is complex, which depends on various factors, including the patient's baseline disease burden and overall clinical picture, not solely on the risk of severe COVID-19 pneumonia. Thus, our model and nomogram can make assistance for doctors by providing an objective and quantifiable estimate of the probability progressed to severe COVID-19 pneumonia.

Our study still has several limitations. First, our nomogram does not include other important predictive variables including heart diseases, chronic obstructive pulmonary disease (COPD), and CT images because of relatively small cohort population. Second, multi-organ dysfunctions of patients with severe COVID-19 pneumonia were evaluated by laboratory examinations of indicated indexes, which needs further pathological validations by biopsy specimens. Third, follow-up data were not available for majority of patients, we could not monitor the risk change of patients during the whole illness onset. Finally, although we have validated our nomogram in an independent cohort, large-scale cohort validation and long-term follow-up are still needed to confirm our findings. Therefore, our nomogram may be improved as additional predictive variables are incorporated, and it still needs follow-up data to accurately monitor the disease progression of COVID-19 pneumonia.

## Conclusion

In conclusion, our study summarized all the existing literatures (published or preprinted), which studied the factors of disease progression of COVID-19 pneumonia. Severe COVID-19 pneumonia is significantly correlated with severe complications such as ARDS, shock, acute kidney injury and acute cardiac injury, and the comorbidities significantly increased the risk of progressive COVID-19 pneumonia. At the same time, a model followed by a nomogram based on previously identified risk factors including age, fever, comorbidities, CK, LDH, CREA, BUN and neutrophil count for predicting the risk of severe COVID-19 pneumonia was established in a development cohort, and further validated in an independent cohort.

## Data Availability Statement

All datasets presented in this study are included in the article/supplementary material.

## Ethics Statement

The study is approved by the Ethics Committee of the Zhongnan Hospital of Wuhan University and the First hospital of Xiaoshan District under the accession 2020035. The patients/participants provided their written informed consent to participate in this study.

## Author Contributions

GC, PL, and YC contributed equally as the co-author. QS and MG had full access to all of the data in the study and take responsibility for the integrity of the data and the accuracy of the data analysis. GC, MG, RZ, and QS concept and design. GC, PL, KF, YC, SW, BC, XF, and QS acquisition, analysis, or interpretation of data. GC, PL, and QS drafting of the manuscript. MX and MG critical revision of the manuscript for important intellectual content. GC and QS statistical analysis. RZ and YC administrative, technical, or material support. MX, RZ, MG, and QS supervision. MG obtain funding. All authors contributed to the article and approved the submitted version.

## Conflict of Interest

The authors declare that the research was conducted in the absence of any commercial or financial relationships that could be construed as a potential conflict of interest.
