# Supplementary material for: A Risk Prediction Model for Evaluating the Disease Progression of COVID-19 Pneumonia
Source: Front Med (Lausanne). 2020 Nov 5;7:556886. doi: 10.3389/fmed.2020.556886 (PMC7675774; doi:10.3389/fmed.2020.556886)
Supplement: Supplementary file 1 [file Data_Sheet_1.docx]

**Supplementary information**

**A Risk Prediction Model for Evaluating the disease progression of COVID-19 pneumonia**

Guodong Cao^1,2,#^, Pengping Li^3,#^, Yuanyuan Chen^4^, Kun Fang^5^, Bo Chen^1^, Shuyue Wang^2^, Xudong Feng^2^, Zhenyu Wang^3^, Maoming Xiong^1,*^, Ruiying Zheng^6,*^, Mengzhe Guo^7,*^, Qiang Sun^7,*^

**Supplementary Text S1** detailed methods

***Meta-analyses***

***Search strategy***

Relevant articles were searched by two investigators independently in the PubMed, Web of Science, and CNKI databases, from inception to Mar 18, 2020. The search terms included ("COVID-19" OR "SARS-CoV-2" OR "2019 novel coronavirus ") AND ("Clinical data" OR "Clinical features" OR "Clinical characteristics"). Full texts were reviewed to assess whether the papers met the inclusion criteria.

***The inclusive and exclusive criteria***

Studies were considered to be relevant if they met the following criteria: (1) provided the clinical features of COVID-19 patients; (2) the hazard ratio (HR) of risk factors were showed; (3) English and Chinese as publication language. Exclusion criteria were: (1) data repetition; (2) reviews; (3) case reports; (4) erroneous data.

***Data extraction and quality evolution***

According to selection criteria, all relevant data were extracted from each eligible study independently by two investigators (Guodong Cao and Pengping Li). Disagreement was resolved by team discussion until consensus was reached. The extracted data included first author’s name, year of publication, total number of patients, clinicopathological parameters, HR values and clinical outcomes. Two investigators independently evaluated the quality of eligible studies by the Newcastle- Ottawa scale.

***Meta-analysis statistics***

Statistical analyses were performed with the STATA software (version 12.0, StataCorp LP, College Station, TX, USA). Crude odds ratios (ORs) and 95% conﬁdence intervals (CIs) were used to estimate the strength of the association between clinical features and COVID-19. ORs/risk ratios (RRs) and 95%CIs were used to estimate the relationship of complications/death and severe COVID-19. Standardized mean difference (SMD) and 95%CIs were used to estimate the relationship between lab findings and severe COVID-19 when data were expressed as mean±SD. The *I*^2^ index, which indicates the percentage of the total variation across studies, was used to assess statistical heterogeneity. A random-effects model was used in case of significant heterogeneity (*I*^2^ > 50% or *P* < 0.1); otherwise, a fixed-effects model was employed. To assess potential publication bias, the Begg's rank correlation test and Egger's weighted regression method was used, with *P* < 0.05 indicating statistically significant publication bias.

**Supplementary Text S2** PRISMA Checklist: Preferred Reporting Items for Systematic Reviews and Meta-Analyses

| **Section/topic** | **#** | **Checklist item** | **Reported on page #** |  |  |
| --- | --- | --- | --- | --- | --- |
| **TITLE** | | |  |  |  |
| Title | 1 | Identify the report as a systematic review, meta-analysis, or both. | Page 1 |  |  |
| **ABSTRACT** | | |  |  |  |
| Structured summary | 2 | Provide a structured summary including, as applicable: background; objectives; data sources; study eligibility criteria, participants, and interventions; study appraisal and synthesis methods; results; limitations; conclusions and implications of key findings; systematic review registration number. | Page 4 |  |  |
| **INTRODUCTION** | | |  |  |  |
| Rationale | 3 | Describe the rationale for the review in the context of what is already known. | Page 5-6 |  |  |
| Objectives | 4 | Provide an explicit statement of questions being addressed with reference to participants, interventions, comparisons, outcomes, and study design (PICOS). | Page 6 |  |  |
| **METHODS** | | |  |  |  |
| Protocol and registration | 5 | Indicate if a review protocol exists, if and where it can be accessed (e.g., Web address), and, if available, provide registration information including registration number. | Appendix page 2 |  |  |
| Eligibility criteria | 6 | Specify study characteristics (e.g., PICOS, length of follow-up) and report characteristics (e.g., years considered, language, publication status) used as criteria for eligibility, giving rationale. | Appendix page 2 |  |  |
| Information sources | 7 | Describe all information sources (e.g., databases with dates of coverage, contact with study authors to identify additional studies) in the search and date last searched. | Appendix page 2 |  |  |
| Search | 8 | Present full electronic search strategy for at least one database, including any limits used, such that it could be repeated. | Appendix page 2 |  |  |
| Study selection | 9 | State the process for selecting studies (i.e., screening, eligibility, included in systematic review, and, if applicable, included in the meta-analysis). | Appendix page 2 |  |  |
| Data collection process | 10 | Describe method of data extraction from reports (e.g., piloted forms, independently, in duplicate) and any processes for obtaining and confirming data from investigators. | Appendix page 2 |  |  |
| Data items | 11 | List and define all variables for which data were sought (e.g., PICOS, funding sources) and any assumptions and simplifications made. |  |  |  |
| Risk of bias in individual studies | 12 | Describe methods used for assessing risk of bias of individual studies (including specification of whether this was done at the study or outcome level), and how this information is to be used in any data synthesis. | Appendix page 2 |  |  |
| Summary measures | 13 | State the principal summary measures (e.g., risk ratio, difference in means). |  |  |  |
| Synthesis of results | 14 | Describe the methods of handling data and combining results of studies, if done, including measures of consistency (e.g., I^2^) for each meta-analysis. | Appendix page 2 |  |  |

| **Section/topic** | **#** | **Checklist item** | **Reported on page #** |  |  |
| --- | --- | --- | --- | --- | --- |
| Risk of bias across studies | 15 | Specify any assessment of risk of bias that may affect the cumulative evidence (e.g., publication bias, selective reporting within studies). |  |  |  |
| Additional analyses | 16 | Describe methods of additional analyses (e.g., sensitivity or subgroup analyses, meta-regression), if done, indicating which were pre-specified. |  |  |  |
| **RESULTS** | | |  |  |  |
| Study selection | 17 | Give numbers of studies screened, assessed for eligibility, and included in the review, with reasons for exclusions at each stage, ideally with a flow diagram. | Page 6 |  |  |
| Study characteristics | 18 | For each study, present characteristics for which data were extracted (e.g., study size, PICOS, follow-up period) and provide the citations. |  |  |  |
| Risk of bias within studies | 19 | Present data on risk of bias of each study and, if available, any outcome level assessment (see item 12). | Page 8 |  |  |
| Results of individual studies | 20 | For all outcomes considered (benefits or harms), present, for each study: (a) simple summary data for each intervention group (b) effect estimates and confidence intervals, ideally with a forest plot. | Page8-10,  appendix  page 5-7 |  |  |
| Synthesis of results | 21 | Present results of each meta-analysis done, including confidence intervals and measures of consistency. |  |  |  |
| Risk of bias across studies | 22 | Present results of any assessment of risk of bias across studies (see Item 15). |  |  |  |
| Additional analysis | 23 | Give results of additional analyses, if done (e.g., sensitivity or subgroup analyses, meta-regression [see Item 16]). |  |  |  |
| **DISCUSSION** | | |  |  |  |
| Summary of evidence | 24 | Summarize the main findings including the strength of evidence for each main outcome; consider their relevance to key groups (e.g., healthcare providers, users, and policy makers). | Page  11-12 |  |  |
| Limitations | 25 | Discuss limitations at study and outcome level (e.g., risk of bias), and at review-level (e.g., incomplete retrieval of identified research, reporting bias). | Page 14 |  |  |
| Conclusions | 26 | Provide a general interpretation of the results in the context of other evidence, and implications for future research. | Page 15 |  |  |
| **FUNDING** | | |  |  |  |
| Funding | 27 | Describe sources of funding for the systematic review and other support (e.g., supply of data); role of funders for the systematic review. |  |  |  |

**Supplementary TABLE S1** analysis of association between complications/death and disease progression of patients with COVID-19 pneumonia

| **Complications and death** | **Studies** | **Total cases** | ***I^2^(%)*** | **Model** | **OR/RR** | **95%CI** | ***P* value** |
| --- | --- | --- | --- | --- | --- | --- | --- |
| ARDS | 10 | 2246 | 69.3 | RE | 49.03 | 20.14-119.35 | <0.001 |
| Shock | 9 | 2135 | 46.8 | FE | 45.48 | 19.85-104.18 | <0.001 |
| Acute kidney injury | 7 | 1787 | 8.9 | FE | 24.82 | 11.40-54.02 | <0.001 |
| Acute cardiac injury | 8 | 1015 | 34.7 | FE | 37.93 | 17.92-80.28 | <0.001 |
| Arrhythmia | 2 | 359 | 68.7 | RE | 20.82 | 9.34-46.41 | <0.001 |
| Secondary infection | 4 | 317 | 36.5 | FE | 32.69 | 12.69-84.27 | <0.001 |
| DIC | 3 | 1446 | 0 | FE | 14.53 | 1.50-140.94 | 0.021 |
| Rhabdomyolysis | 2 | 1179 | 0 | FE | 1.07 | 0.05-22.29 | 0.967 |
| Heart failure | 2 | 165 | 0 | FE | 9.59 | 1.01-91.12 | 0.049 |
| Liver dysfunction | 2 | 98 | 0 | FE | 7.59 | 2.46-23.39 | <0.001 |
| Death | 5 | 1918 | 26.2 | FE | 30.09 | 11.46-79.01 | <0.001 |

**Supplementary TABLE S2** Meta-analysis of association between clinical characteristics and disease progression of patients with COVID-19 pneumonia

| **Categories** | **Parameters** | **Studies** | **Total cases** | ***I^2^*(%)** | **Model** | **OR** | **95%CI** | ***P* value** |
| --- | --- | --- | --- | --- | --- | --- | --- | --- |
| **Basic information** | Gender | 29 | 4966 | 26.6 | FE | 1.57 | 1.36-1.80 | <0.001 |
|  | Exposure history | 14 | 1853 | 41.1 | FE | 0.99 | 0.78-1.30 | 0.958 |
|  | Smoking | 12 | 2621 | 73.4 | RE | 2.10 | 1.04-4.24 | 0.039 |
|  | Alcohol | 2 | 353 | 0 | FE | 0.76 | 0.18-3.19 | 0.709 |
| **Comorbidities** | Hypertension | 26 | 4912 | 57.6 | RE | 2.53 | 1.89-3.37 | <0.001 |
|  | Diabetes | 28 | 5047 | 34.6 | FE | 2.43 | 1.98-2.97 | <0.001 |
|  | Cancer | 15 | 2987 | 20.4 | FE | 1.73 | 1.07-2.78 | 0.025 |
|  | Heart diseases | 21 | 4430 | 46.2 | FE | 4.11 | 3.15-5.35 | <0.001 |
|  | Pulmonary diseases | 18 | 3979 | 0 | FE | 4.17 | 2.86-6.08 | <0.001 |
|  | Kidney diseases | 11 | 2746 | 0 | FE | 5.44 | 2.81-10.54 | <0.001 |
|  | Cerebrovascular disease | 10 | 3004 | 0 | FE | 4.02 | 2.41-6.60 | <0.001 |
|  | Liver diseases | 14 | 2502 | 0 | FE | 1.47 | 0.92-2.35 | 0.104 |
|  | Stomach diseases | 2 | 293 | 28.9 | FE | 0.86 | 0.22-3.39 | 0.832 |
|  | Thyroid diseases | 4 | 531 | 0 | FE | 2.13 | 0.72-6.29 | 0.17 |
|  | Immunosuppression | 5 | 1660 | 0 | FE | 1.19 | 0.36-3.89 | 0.778 |
| **Clinical** **symptoms** | Fever | 23 | 4513 | 42.6 | FE | 1.22 | 1.01-1.47 | 0.043 |
|  | Cough | 26 | 4723 | 26.2 | FE | 1.13 | 0.97-1.32 | 0.117 |
|  | Chest soreness | 7 | 2213 | 28.7 | FE | 0.94 | 0.63-1.40 | 0.75 |
|  | Expectoration | 14 | 2611 | 38.6 | FE | 1.31 | 1.05-1.63 | 0.016 |
|  | Fatigue | 20 | 4320 | 53.7 | RE | 1.47 | 1.11-1.93 | 0.007 |
|  | Muscular soreness | 17 | 2425 | 43.4 | FE | 1.44 | 1.13-1.85 | 0.004 |
|  | Breath problem | 20 | 3844 | 56.1 | RE | 4.27 | 3.07-5.95 | <0.001 |
|  | Abdominal pain, diarrhea | 20 | 4115 | 22.8 | FE | 1.22 | 0.88-1.69 | 0.236 |
|  | Headache or dizziness | 17 | 3415 | 0 | FE | 1.22 | 0.92-1.61 | 0.17 |
|  | Chest congestion | 10 | 1476 | 24.7 | FE | 1.92 | 1.40-2.63 | <0.001 |
|  | Anorexia | 7 | 1426 | 67.7 | RE | 1.76 | 1.00-3.09 | 0.050 |
|  | Pharyngalgia | 10 | 2031 | 53.0 | RE | 0.93 | 0.48-1.80 | 0.837 |
|  | Rhinorrhea | 6 | 1298 | 0 | FE | 0.80 | 0.33-1.93 | 0.617 |
|  | Nausea or vomiting | 8 | 2540 | 53.3 | RE | 2.00 | 0.98-4.09 | 0.057 |
|  | Hemoptysis | 4 | 1685 | 75.5 | RE | 0.62 | 0.07 | 0.659 |

**Supplementary TABLE S3** Meta-analysis of association between laboratory findings and disease progression of patients with COVID-19 pneumonia

| **Categories** | **Parameters** | **Studies** | **Total cases** | **SMD** | **95%CI** | ***P* value** |
| --- | --- | --- | --- | --- | --- | --- |
| **Basic information** | Age | 24 | 4097 | 2.04 | 1.45~2.63 | <0.001 |
| **Blood routine** | Hb | 12 | 2357 | -0.74 | -1.34~-0.13 | 0.018 |
|  | Lymphocyte | 23 | 4125 | -2.35 | -2.85~-1.86 | <0.001 |
|  | WBC | 22 | 3800 | 1.35 | 0.49~2.22 | 0.002 |
|  | Neutrophil | 18 | 2472 | 2.21 | 1.70~2.73 | <0.001 |
|  | Monocyte | 5 | 594 | 1.46 | -0.39~3.31 | 0.122 |
|  | PLT | 18 | 3257 | -0.88 | -1.42~-0.34 | 0.001 |
| **Immunocyte** | CD3 lymphocyte | 2 | 351 | -9.84 | -19.94~0.27 | 0.056 |
|  | CD4 lymphocyte | 6 | 690 | -5.08 | -7.34~-2.82 | <0.001 |
|  | CD8 lymphocyte | 5 | 690 | -4.79 | -7.16~-2.43 | <0.001 |
| **Blood biochemistry** | K^+^ | 7 | 1979 | -0.31 | -1.10~0.48 | 0.445 |
|  | Na^+^ | 7 | 1979 | -1.85 | -3.09~-0.62 | 0.003 |
|  | Ca^+^ | 2 | 489 | -2.32 | -6.90~2.25 | 0.319 |
|  | ALT | 20 | 2968 | 1.29 | 0.84~1.74 | <0.001 |
|  | AST | 17 | 2139 | 2.01 | 1.20~2.81 | <0.001 |
|  | CREA | 21 | 3068 | 1.20 | 0.72~1.68 | <0.001 |
|  | BUN | 13 | 1755 | 2.44 | 1.52~3.36 | <0.001 |
|  | Total bilirubin | 12 | 2171 | 0.88 | 0.06~1.70 | 0.036 |
|  | Albumin | 11 | 1650 | -3.18 | -3.98~-2.38 | <0.001 |
|  | Serum lactate | 4 | 799 | 0.27 | -0.76~1.31 | 0.604 |
| **Coagulation function** | D-dimer | 15 | 2571 | 2.05 | 1.53~2.57 | <0.001 |
|  | Prothrombin time | 11 | 1866 | 1.86 | 1.01~2.70 | <0.001 |
|  | Partial thromboplastin time | 9 | 1237 | 0.36 | -0.92~1.63 | 0.252 |
|  | Fibrinogen | 4 | 804 | 1.89 | -0.33~4.11 | 0.095 |
| **Infection-related parameters** | CRP | 19 | 2656 | 4.8 | 3.23~5.33 | <0.001 |
|  | Procalcitonin | 9 | 1042 | 1.85 | 0.76~2.93 | <0.001 |
|  | ESR | 5 | 897 | 0.96 | 0.63~1.30 | <0.001 |
|  | Ferritin | 5 | 533 | 2.73 | 1.21~4.25 | <0.001 |
| **Myocardial enzyme** | LDH | 16 | 2258 | 4.04 | 2.73~5.36 | <0.001 |
|  | cTnI | 5 | 856 | 1.84 | 0.77~2.91 | 0.001 |
|  | Myoglobin | 2 | 463 | 6.50 | 4.32~8.69 | <0.001 |
|  | CK | 14 | 1793 | 1.64 | 0.71~2.56 | 0.001 |
|  | CK-MB | 5 | 963 | 1.80 | 1.55~2.05 | <0.001 |

**Supplementary TABLE S4** Meta-analysis of the pool Hazard Ratios between potential risk factors and disease progression of patients with COVID-19 pneumonia

| **Parameters** | **Studies** | **HR** | **95% CI** | ***P* value** |
| --- | --- | --- | --- | --- |
| Age | 11 | 1.31 | 1.17-1.47 | <0.001 |
| Sex | 9 | 1.26 | 0.83-1.91 | 0.271 |
| Hypertension | 6 | 2.11 | 1.60-2.79 | <0.001 |
| Diabetes | 8 | 1.71 | 1.16-2.54 | 0.007 |
| Smoking | 2 | 2.19 | 0.55-8.69 | 0.265 |
| Pulmonary diseases | 3 | 3.60 | 1.91-6.78 | 0.006 |
| Heart diseases | 5 | 5.02 | 1.47-17.10 | 0.010 |
| Fever | 3 | 1.73 | 1.06-2.84 | 0.029 |
| Cough | 2 | 2.78 | 0.32-24.39 | 0.355 |
| Respiratory rate | 4 | 3.85 | 0.84-17.61 | 0.082 |
| CT scores | 2 | 8.69 | 1.53-49.50 | 0.015 |
| Blood oxygen saturation | 2 | 0.94 | 0.89-0.99 | 0.025 |
| Hb | 3 | 1.00 | 0.96-1.05 | 0.873 |
| ALT | 5 | 1.43 | 0.98-2.08 | 0.065 |
| AST | 4 | 1.02 | 0.98-1.05 | 0.409 |
| Total bilirubin | 2 | 2.19 | 0.81-5.91 | 0.121 |
| Albumin | 4 | 1.75 | 0.80-3.80 | 0.162 |
| Creatinine | 8 | 1.08 | 1.02-1.15 | 0.007 |
| BUN | 5 | 1.54 | 1.28-1.87 | <0.001 |
| CRP | 7 | 1.02 | 0.99-1.04 | 0.165 |
| Lymphocyte | 7 | 0.73 | 0.16-3.36 | 0.688 |
| ESR | 2 | 1.19 | 0.59-2.40 | 0.632 |
| WBC | 4 | 2.59 | 0.93-7.19 | 0.068 |
| Neutrophils | 5 | 1.17 | 1.00-1.38 | 0.050 |
| Procalcitonin | 4 | 4.07 | 1.05-15.73 | 0.042 |
| IL-6 | 2 | 1.06 | 0.97-1.16 | 0.22 |
| Serum ferritin | 2 | 4.59 | 2.00-10.55 | <0.001 |
| Platelets | 2 | 1.00 | 0.99-1.00 | 0.241 |
| D-dimer | 3 | 3.25 | 0.44-23.79 | 0.246 |
| Prothrombin time | 2 | 2.22 | 0.82-6.00 | 0.117 |
| CK | 2 | 2.13 | 1.44-3.15 | <0.001 |
| CK-MB | 2 | 1.12 | 0.90-1.38 | 0.319 |
| LDH | 4 | 2.15 | 1.32-3.51 | 0.002 |
| cTnI | 5 | 4.87 | 1.48-16.01 | 0.009 |
| NT-proBNP | 2 | 4.00 | 0.43-36.99 | 0.222 |


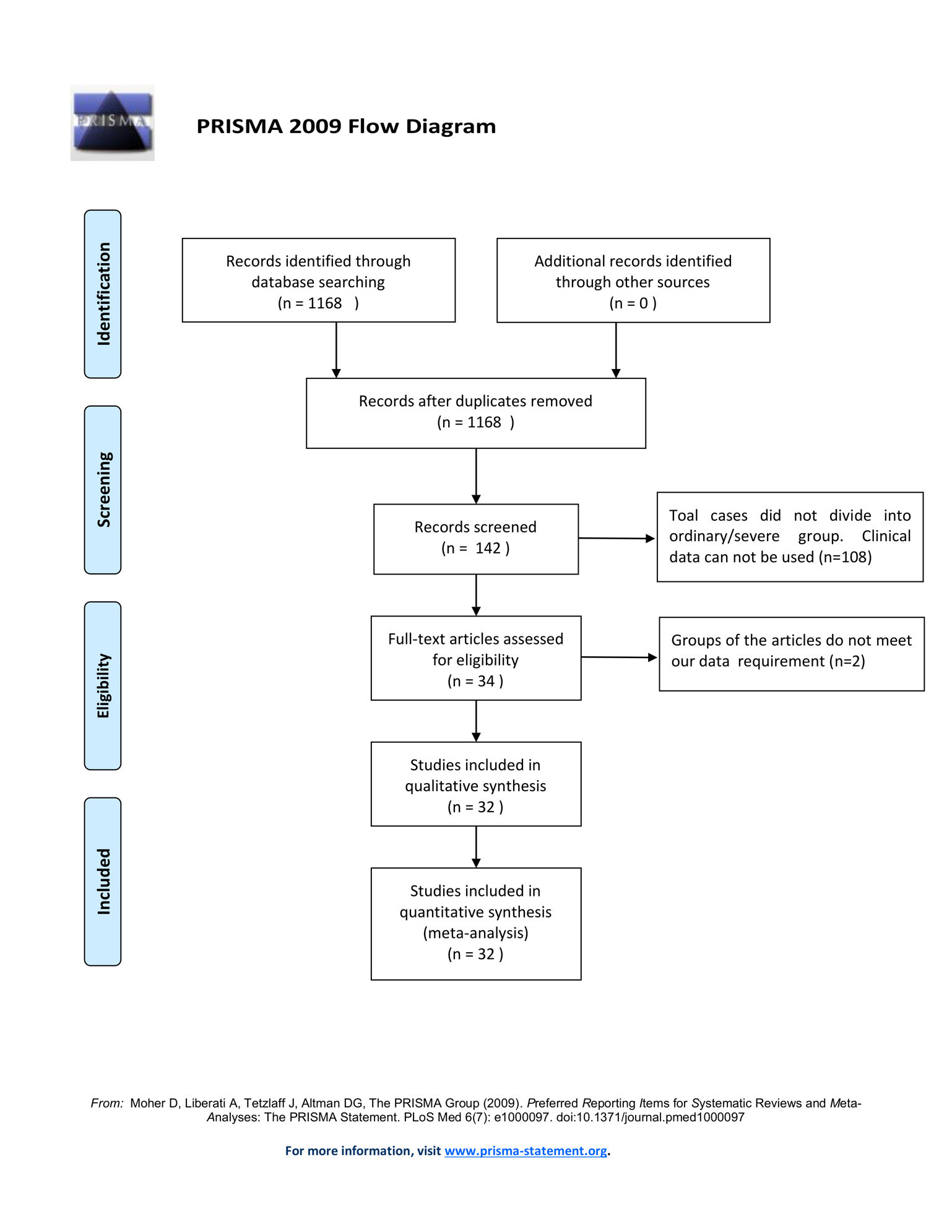
**Supplementary Figure S1** FlowDiagram of the study selection procedure of the meta-analysis

**Supplementary Figure S2** Publication bias: Begg’s (A) (*P* > 0.05) and Egger’s (B) (*P* > 0.05) funnel plots for possible publication bias in the current study. No publication bias was found, indicating credible results.

**
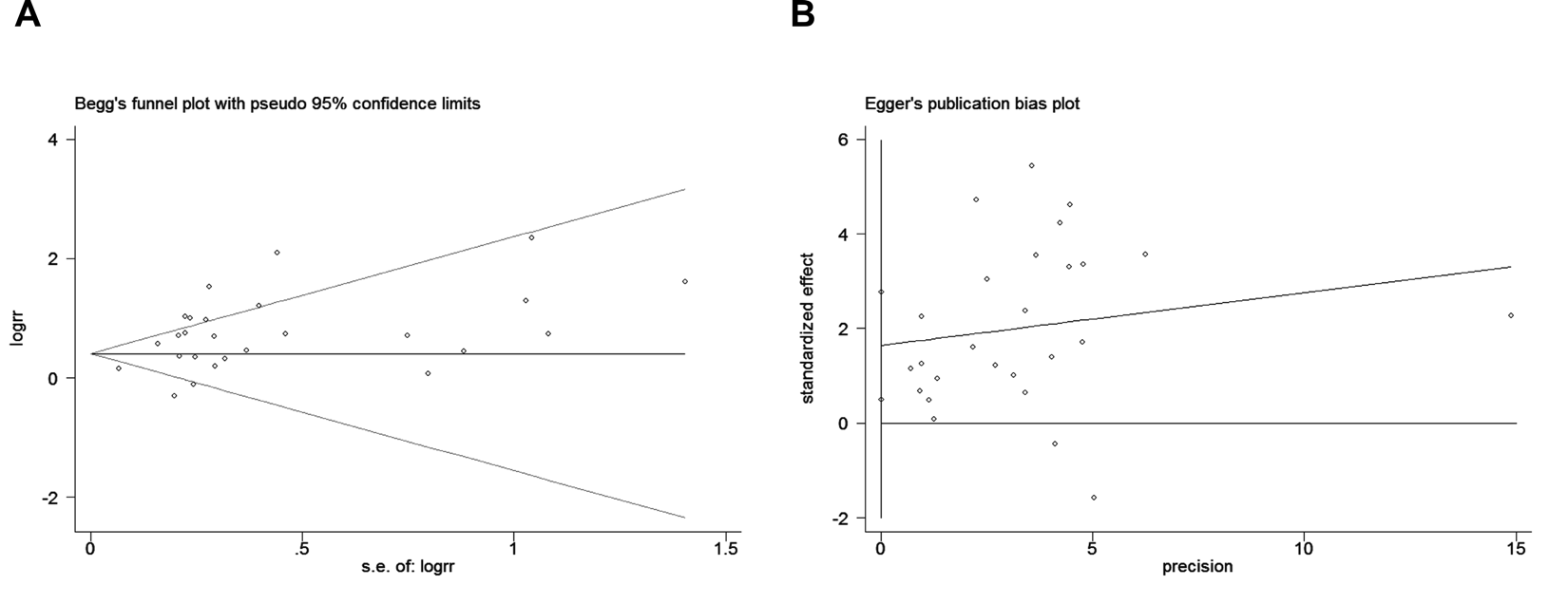
**

**Supplementary Figure S3** Forest plots of standardized mean differences showed the associations between immune cell counts (A-C) or inflammation-related index (D) and disease progression of COVID-19 pneumonia.


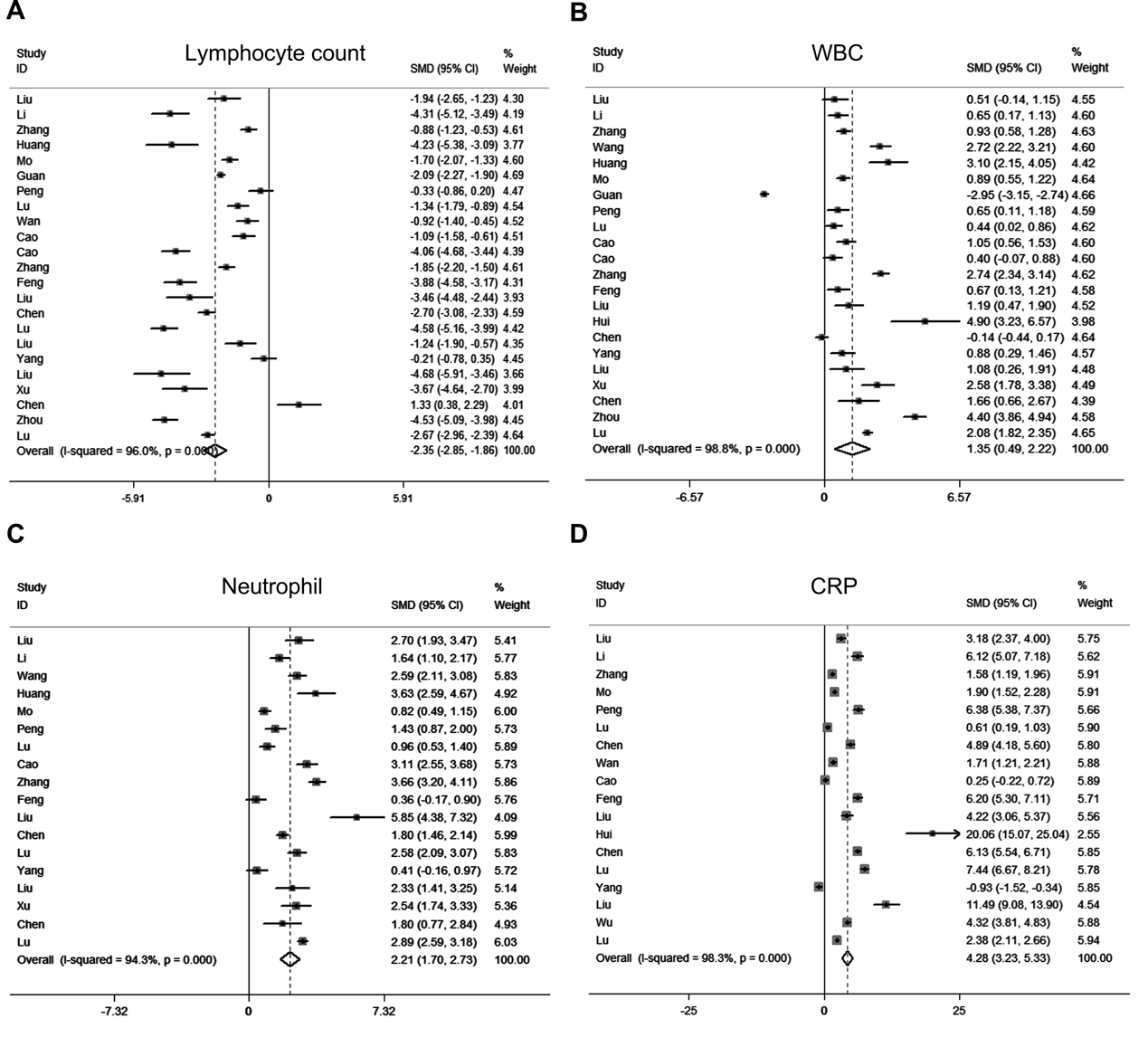


**Supplementary Figure S4** Forest plots of standardized mean differences showed the associations between liver function indexes (A-B), renal function indexes (C-D) as well as heart function indexes (E-F) and disease progression of COVID-19 pneumonia.

**
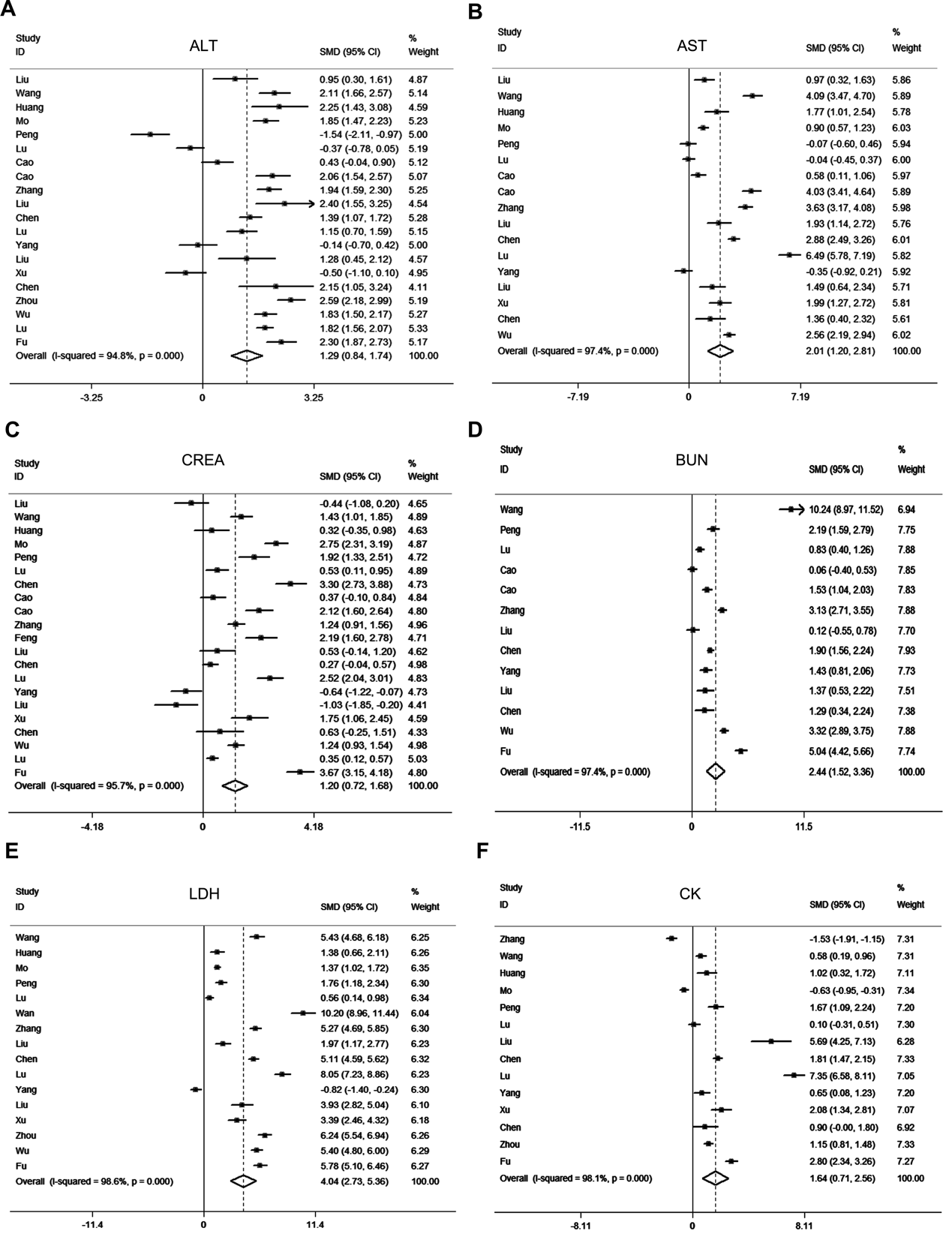
**

**Supplementary Figure S5** Venn diagram represented the overlapped risk factors identified by both meta-analysis and univariate logistic regression analysis

**
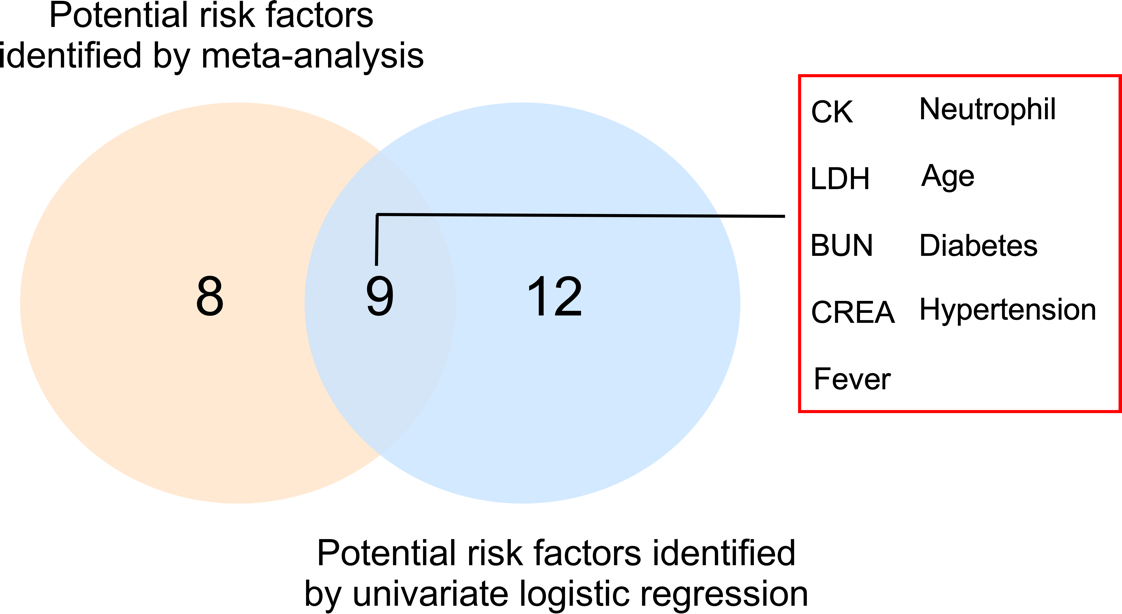
**

**Supplementary Figure S6** (A) Correlation between the laboratory test parameters. (B-D) Receiver operating characteristic curves of sensitivity and specificity of the nomogram for predicting severe COVID-19 pneumonia in development (D), Hangzhou validation (E), and Yinchuan validation cohorts (F).

**
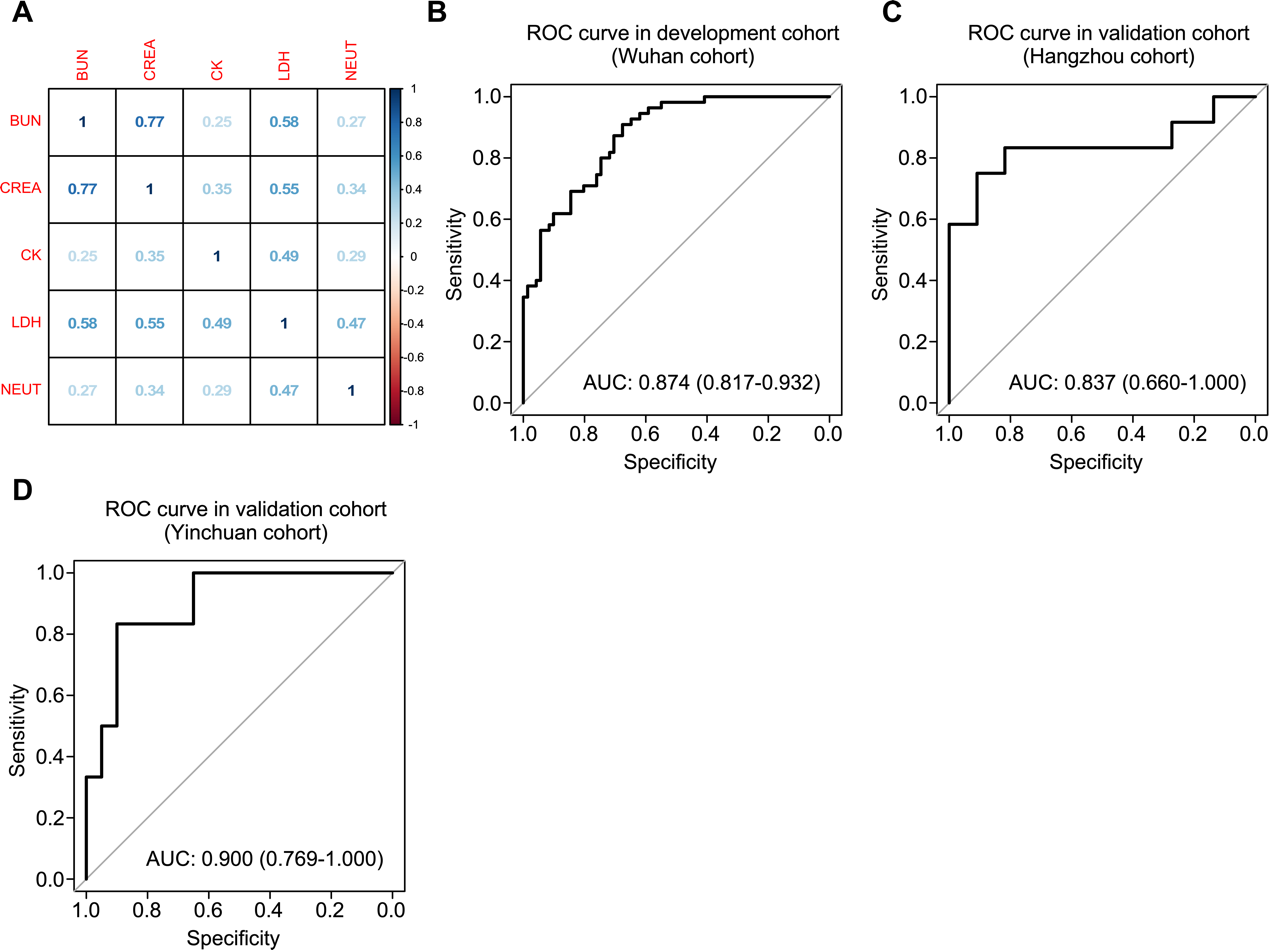
**

**References(1-32)**

1. Liu, W., Tao, Z. W., Lei, W., Ming-Li, Y., Kui, L., Ling, Z., et al. (2020) Analysis of factors associated with disease outcomes in hospitalized patients with 2019 novel coronavirus disease. *Chin Med J (Engl)* doi:10.1097/CM9.0000000000000775

2. Li, K., Wu, J., Wu, F., Guo, D., Chen, L., Fang, Z., et al. (2020) The Clinical and Chest CT Features Associated with Severe and Critical COVID-19 Pneumonia. *Invest Radiol* doi:10.1097/RLI.0000000000000672

3. Zhang, J. J., Dong, X., Cao, Y. Y., Yuan, Y. D., Yang, Y. B., Yan, Y. Q., et al. (2020) Clinical characteristics of 140 patients infected with SARS-CoV-2 in Wuhan, China. *Allergy* doi:10.1111/all.14238

4. Wang, D., Hu, B., Hu, C., Zhu, F., Liu, X., Zhang, J., et al. (2020) Clinical Characteristics of 138 Hospitalized Patients With 2019 Novel Coronavirus-Infected Pneumonia in Wuhan, China. *JAMA* doi:10.1001/jama.2020.1585

5. Huang, C., Wang, Y., Li, X., Ren, L., Zhao, J., Hu, Y., et al. (2020) Clinical features of patients infected with 2019 novel coronavirus in Wuhan, China. *Lancet* 395 (10223), 497-506. doi:10.1016/S0140-6736(20)30183-5

6. Mo, P., Xing, Y., Xiao, Y., Deng, L., Zhao, Q., Wang, H., et al. (2020) Clinical characteristics of refractory COVID-19 pneumonia in Wuhan, China. *Clin Infect Dis* doi:10.1093/cid/ciaa270

7. Guan, W. J., Ni, Z. Y., Hu, Y., Liang, W. H., Ou, C. Q., He, J. X., et al. (2020) Clinical Characteristics of Coronavirus Disease 2019 in China. *N Engl J Med* doi:10.1056/NEJMoa2002032

8. Peng, Y. D., Meng, K., Guan, H. Q., Leng, L., Zhu, R. R., Wang, B. Y., et al. (2020) Clinical characteristics and outcomes of 112 cardiovascular disease patients infected by 2019-nCoV. *Zhonghua Xin Xue Guan Bing Za Zhi* 48 (0), E004. doi:10.3760/cma.j.cn112148-20200220-00105

9. Lu, Z., He, R., Jiang, W., Fan, T., and Geng, Q. (2020) Clinical characteristics and immune function analysis of COVID‐19. *Medical Journal of Wuhan University* doi:10.14188/j.1671‐8852.2020.0126

10. Chen, C., Chen, C., Yan, J. T., Zhou, N., Zhao, J. P., and Wang, D. W. (2020) Analysis of myocardial injury in patients with COVID-19 and association between concomitant cardiovascular diseases and severity of COVID-19. *Zhonghua Xin Xue Guan Bing Za Zhi* 48 (0), E008. doi:10.3760/cma.j.cn112148-20200225-00123

11. Wan, Q., Shi, A., He, T., and tang, L. (2020) Analysis of clinical features of 153 patients with novel coronavirus pneumonia in Chongqing. *Chinese Journal of Clinical Infectious Diseases* doi:10.3760/cma.j.cn 115673-20200212-00030

12. Zhou, F., Yu, T., Du, R., Fan, G., Liu, Y., Liu, Z., et al. (2020) Clinical course and risk factors for mortality of adult inpatients with COVID-19 in Wuhan, China: a retrospective cohort study. *The Lancet* 395 (10229), 1054-1062. doi:10.1016/s0140-6736(20)30566-3

13. Wu, C., Chen, X., Cai, Y., Xia, J., Zhou, X., Xu, S., et al. (2020) Risk Factors Associated With Acute Respiratory Distress Syndrome and Death in Patients With Coronavirus Disease 2019 Pneumonia in Wuhan, China. *JAMA Intern Med* doi:10.1001/jamainternmed.2020.0994

14. Cao, W., Shi, L., Chen, L., Xu, X., and Wu, Z. (2020) Clinical features and laboratory inspection of novel coronavirus pneumonia (COVID-19) in Xiangyang, Hubei. *medRxiv* doi:10.1101/2020.02.23.20026963

15. Cao, M., Zhang, D., Wang, Y., Lu, Y., Zhu, X., Li, Y., et al. (2020) Clinical Features of Patients Infected with the 2019 Novel Coronavirus (COVID-19) in Shanghai, China. *medRxiv* doi:10.1101/2020.03.04.20030395

16. Zhang, G., Hu, C., Luo, L., Fang, F., Chen, Y., Li, J., et al. (2020) Clinical features and outcomes of 221 patients with COVID-19 in Wuhan, China. *medRxiv* doi:10.1101/2020.03.02.20030452

17. Feng, Z., Yu, Q., Yao, S., Luo, L., Duan, J., Yan, Z., et al. (2020) Prediction of Disease Progression in 2019 Novel Coronavirus Pneumonia Patients Outside Wuhan with CT and Clinical Characteristics. *medRxiv* doi:10.1101/2020.02.19.20025296

18. Liu, J., Li, S., Liu, J., Liang, B., Wang, X., Wang, H., et al. (2020) Longitudinal characteristics of lymphocyte responses and cytokine profiles in the peripheral blood of SARS-CoV-2 infected patients. *medRxiv* doi:10.1101/2020.02.16.20023671

19. Chen, X., Zheng, F., Qing, Y., Ding, S., Yang, D., Lei, C., et al. (2020) Epidemiological and clinical features of 291 cases with coronavirus disease 2019 in areas adjacent to Hubei, China: a double-center observational study. *medRxiv* doi:10.1101/2020.03.03.20030353

20. Lu, H., Ai, J., Shen, Y., Li, Y., Li, T., Zhou, X., et al. (2020) A descriptive study of the impact of diseases control and prevention on the epidemics dynamics and clinical features of SARS-CoV-2 outbreak in Shanghai, lessons learned for metropolis epidemics prevention. *medRxiv* doi:10.1101/2020.02.19.20025031

21. Liu, T., Zhang, J., Yang, Y., Ma, H., Li, Z., Zhang, J., et al. (2020) The potential role of IL-6 in monitoring severe case of coronavirus disease 2019. *medRxiv* doi:10.1101/2020.03.01.20029769

22. Yang, Y., Shen, C., Li, J., Yuan, J., Yang, M., Wang, F., et al. (2020) Exuberant elevation of IP-10, MCP-3 and IL-1ra during SARS-CoV-2 infection is associated with disease severity and fatal outcome. *medRxiv* doi:10.1101/2020.03.02.20029975

23. Liu, L., Gao, J., Hu, W., Zhang, X., Guo, L., Liu, C., et al. (2020) Clinical characteristics of 51 patients discharged from hospital with COVID-19 in Chongqing, China. *medRxiv* doi:10.1101/2020.02.20.20025536

24. Xu, Y., Xu, Z., Liu, X., Cai, L., Zheng, H., Huang, Y., et al. (2020) Clinical findings in critically ill patients infected with SARS-CoV-2 in Guangdong Province, China: a multi-center, retrospective, observational study. *medRxiv* doi:10.1101/2020.03.03.20030668

25. Qi, D., Yan, X., Tang, X., Peng, J., Yu, Q., Feng, L., et al. (2020) Epidemiological and clinical features of 2019-nCoV acute respiratory disease cases in Chongqing municipality, China: a retrospective, descriptive, multiple-center study. *medRxiv* doi:10.1101/2020.03.01.20029397

26. Wang, L., Li, X., Chen, H., Yan, S., Li, Y., Li, D., et al. (2020) SARS-CoV-2 infection does not significantly cause acute renal injury: an analysis of 116 hospitalized patients with COVID-19 in a single hospital, Wuhan, China. *medRxiv* doi:10.1101/2020.02.19.20025288

27. Chen, G., Wu, D., Guo, W., Cao, Y., Huang, D., Wang, H., et al. (2020) Clinical and immunologic features in severe and moderate forms of Coronavirus Disease 2019. *medRxiv* doi:10.1101/2020.02.16.20023903

28. Lu, J., Hu, S., Fan, R., Liu, Z., Yin, X., Wang, Q., et al. (2020) ACP risk grade: a simple mortality index for patients with confirmed or suspected severe acute respiratory syndrome coronavirus 2 disease (COVID-19) during the early stage of outbreak in Wuhan, China. *medRxiv* doi:10.1101/2020.02.20.20025510

29. Shi, Q., Zhao, K., Yu, J., Feng, J., Zhao, K., Zhang, X., et al. (2020) Clinical characteristics of 101 non-surviving hospitalized patients with COVID-19—A single center, retrospective study. *medRxiv* doi:10.1101/2020.03.04.20031039

30. Fu, L., Fei, J., Xiang, H.-X., Xiang, Y., Tan, Z.-X., Li, M.-D., et al. (2020) Influence factors of death risk among COVID-19 patients in Wuhan, China: a hospital-based case-cohort study. *medRxiv* doi:10.1101/2020.03.13.20035329

31. Cheng, Y., Luo, R., Wang, K., Zhang, M., Wang, Z., Dong, L., et al. (2020) Kidney impairment is associated with in-hospital death of COVID-19 patients. *medRxiv* doi:10.1101/2020.02.18.20023242

32. Gao, L., Jiang, D., Wen, X., Cheng, X., Sun, M., He, B., et al. (2020) Prognostic value of NT-proBNP in patients with severe COVID-19. *medRxiv* doi:10.1101/2020.03.07.20031575
